# Burden of subarachnoid hemorrhage in Asia and ARIMA model prediction trends from 1990 to 2021

**DOI:** 10.1038/s41598-025-18756-7

**Published:** 2025-09-29

**Authors:** Yuntao Li, Minmin Wu, Luwen Zhu

**Affiliations:** 1 Heilongjiang University of Traditional Chinese Medicine, Harbin, China; 2 Rehabilitation Center, The Second Affiliated Hospital of Heilongjiang, University of Chinese Medicine, Harbin, China

**Keywords:** Stroke, Subarachnoid haemorrhage, Asia, Global burden of disease, Neuroscience, Neurological disorders

## Abstract

Subarachnoid hemorrhage (SAH) is a rare but serious stroke subtype that accounts for approximately 5% of all strokes. This study analyzed SAH epidemiology in Asia from 1990 to 2021 using the Global Burden of Disease (GBD) 2021 data. This study assessed the burden of SAH in Asia using four indicators: incidence, prevalence, mortality, and disability-adjusted life years (DALYs). An autoregressive integrated moving average (ARIMA) model was used to conduct a long-term trend analysis and forecast the changing trends of various indicators from 2022 to 2041. Between 1990 and 2021, the age-standardized incidence rates (ASIR) of SAH in Asia increased and then decreased, while the age-standardized prevalence rates (ASPR), age-standardized mortality rates (ASMR), and age-standardized disability-adjusted life years rates (ASDR) of SAH continued to decline. The Arima model predictions suggest that Asian age-standardized rates will typically show a decreasing trend over the next 20 years, except for the ASIR, which shows an increasing trend. Over the next 20 years, the ASIR of SAH in Asia is expected to increase. The ASPR, ASMR, and ASDR were expected to show decreasing trends. The burden of SAH in older patients remains high, highlighting the need to strengthen the health management of the older population.

## Introduction

Subarachnoid hemorrhage (SAH) is defined as bleeding in the subarachnoid space between the arachnoid and pia mater. It accounts for only 5% of all stroke cases and occurs at a relatively young age^1^. Despite its low incidence, when it does occur, the consequences can be extremely severe, resulting in very high rates of disability and mortality^2^. The most common clinical manifestation of spontaneous SAH is sudden onset of unexpected and intense headaches^3^. Survivors often have cognitive impairments. This significantly impacts their ability to engage in daily activities and social interactions, leading to a substantial decline in their quality of life. Many patients remain unable to resume their normal work and lifestyle even after receiving treatment. One study suggested that smoking and hypertension may influence the incidence of SAH^4^. These risk factors are prevalent in the population and, if left unchecked and unaddressed, will increase the risk of SAH for more individuals. SAH imposes an additional financial burden on the Health system, affecting the 50−60-year-old population, and leading to longer hospital stays^5^. This financial burden may divert medical resources away from other diseases, negatively impacting the operational efficiency of the entire healthcare system. This, in turn, has a cascading effect on the overall healthy development of society. In summary, SAH, a type of stroke associated with high rates of disability and mortality, presents a significant public health challenge due to its clinical manifestations, the influence of risk factors, and its economic burden. It is imperative that this issue receives widespread attention and focus from all sectors of the community, and that effective preventive and therapeutic measures are implemented to address this complex problem.

Existing studies have focused on the global burden of stroke^6,7^; however, the burden of SAH has not been adequately studied. This study utilized the latest Global Burden of Disease (GBD) database to assess the burden of SAH in Asia, with a focus on comprehensive regional-level analyses. It analyzes the incidence, prevalence, mortality, and disability-adjusted life years (DALYs) of SAH in Asia from 1990 to 2021, aiming to enhance understanding of its epidemiology and support authorities in identifying gaps in prevention, management, and intervention programs.

## Methods

GBD began 30 years ago to provide timely, valid, and relevant assessments of key health outcomes. For continuous quality improvement, each annual GBD study re-estimates the entire time series by including all known advances in data, modelling, estimation methods, and health literacy, thereby ensuring that each GBD contains the most up-to-date estimates^8^.

The GBD 2021 indicators include estimates and their 95% uncertainty intervals (UI). According to the GBD algorithm, defined by the 25th and 975th ordered values of 1000 draws of the posterior distribution, all ratios were reported for every 100,000 persons^9^. UIs account for not only the variance in parameter estimation but also the uncertainty from data collection, model selection, and other sources of uncertainty under the parameter estimation process^8^.

We used the most recent data from the Global Health Data Exchange (https://vizhub.healthdat.sgbd-results/). Four Asian regions were extracted for the analysis of the burden of SAH: East Asia, South Asia, Southeast Asia, and Central Asia. In addition, we analyzed the burden of SAH in the high-income Asian Pacific.

DALYs represent the sum of years lived with disability (YLD) and years of life lost (YLL). ASPR were used to assess temporal trends in prevalence over the study period.

The autoregressive integrated moving average (ARIMA) (p, d, q) model was applied to forecast trends in the four age-standardized indicators of SAH in Asia from 2022 to 2041. The letters p, d, and q represent the order of autoregression, the degree of difference, and the order of moving average^10^. The best ARIMA (p, d, q) models were selected using the Akaike information criterion (AIC) and Bayesian information criterion (BIC) to predict SAH disease burden. The Ljung–Box Q test was used to determine whether the residuals of the selected models satisfied an independent normal distribution^11^.

This study used the R statistical program (version 4.2.2) for graphical analysis.

## Results

### Burden and Temporal trends of SAH in Asia

**Fig. 1:**
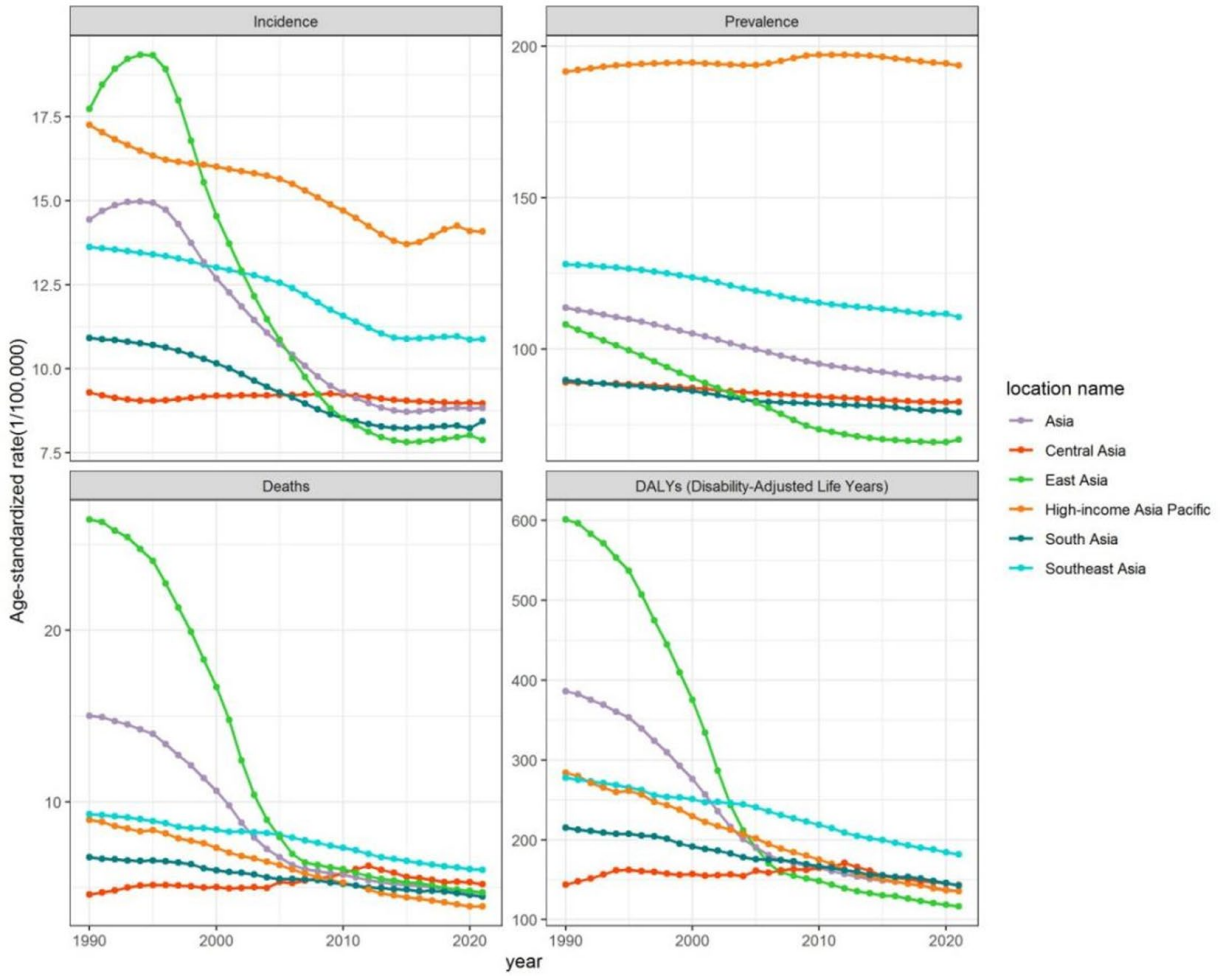
Trends of ASIR, ASPR, ASMR, and ASDR of SAH in Asia, Central Asia, East Asia, South Asia, Southeast Asia, and High-income Asia Pacific from 1990 to 2021. DALY: Disability-adjusted life year; ASIR: Age-standardized incidence rates; ASPR: Age-standardized prevalence rates; ASDR: Age-standardized DALY rates; ASMR: Age-standardized mortality rates.

Table 1 shows the numbers and age-standardized rates of the four SAH indicators in Asia. As shown in Table 1; Fig. 1, the age-standardized incidence rate (ASIR), age-standardized prevalence rates (ASPR), age-standardized mortality rates (ASMR), and age-standardized disability-adjusted life years rate (ASDR) of SAH in Asia declined from 1990 to 2021 by 38.85%, 20.67%, 68.84%, and 64.87%, respectively. In Asia, the ASIR of SAH initially increased, then decreased, and eventually leveled off. The ASPR of SAH showed a flat and decreasing trend, while the ASMR and ASDR of SAH generally showed a sharp decline followed by a more gradual decrease. The ASMR and ASDR of SAH in Asia began to decline at an accelerated rate in 1995, indicating that disease management for SAH likely improved during that year.

In 2021, among the four regions of Asia, Southeast Asia had the highest ASIR, ASPR, ASMR, and ASDR of SAH in all four regions. East Asia had the lowest ASIR, ASPR, and ASDR of SAH, while South Asia had the lowest ASMR of SAH. The high-income Asia-Pacific region had a high ASIR and ASPR for SAH, and a low ASMR for SAH in 2021.

**Table 1:** Disease burden of SAH in Asia in 1990 and 2021.

	1990	2021
Incidence		
Number of cases (95% UI)	325547.23(280372.63, 378757.42)	437719.00(384107.76, 503982.62)
Age-standardized Rate(95% UI) per 100,000	14.44(12.55,16.80)	8.83(7.76, 10.09)
Percentage change of ASR, 1990–2021 (%)	−38.85%	
Prevalence		
Number of cases (95% UI)	2793359.82(2482934.89, 3097698.40)	4597522.40(4163475.01, 5075403.49)
Age-standardized Rate(95% UI) per 100,000	113.65(101.32, 126.86)	90.16(81.67, 99.22)
Percentage change of ASR, 1990–2021 (%)	−20.67%	
Deaths		
Number of cases (95% UI)	282018.69(176374.91, 368350.41)	227499.52(190847.78, 265471.85)
Age-standardized Rate(95% UI) per 100,000	15.02(9.11, 19.82)	4.68(3.90, 5.45)
Percentage change of ASR, 1990–2021 (%)	−68.84%	
DALYs (Disability-Adjusted Life Years)		
Number of cases (95% UI)	8718447.05(6047246.73, 11040941.86)	6853937.02(5831573.20, 7865790.94)
Age-standardized Rate(95% UI) per 100,000	386.03(258.79, 494.39)	135.60(115.64, 155.39)
Percentage change of ASR, 1990–2021 (%)	−64.87%	

### Population distribution of SAH in Asia

**Fig. 2:**
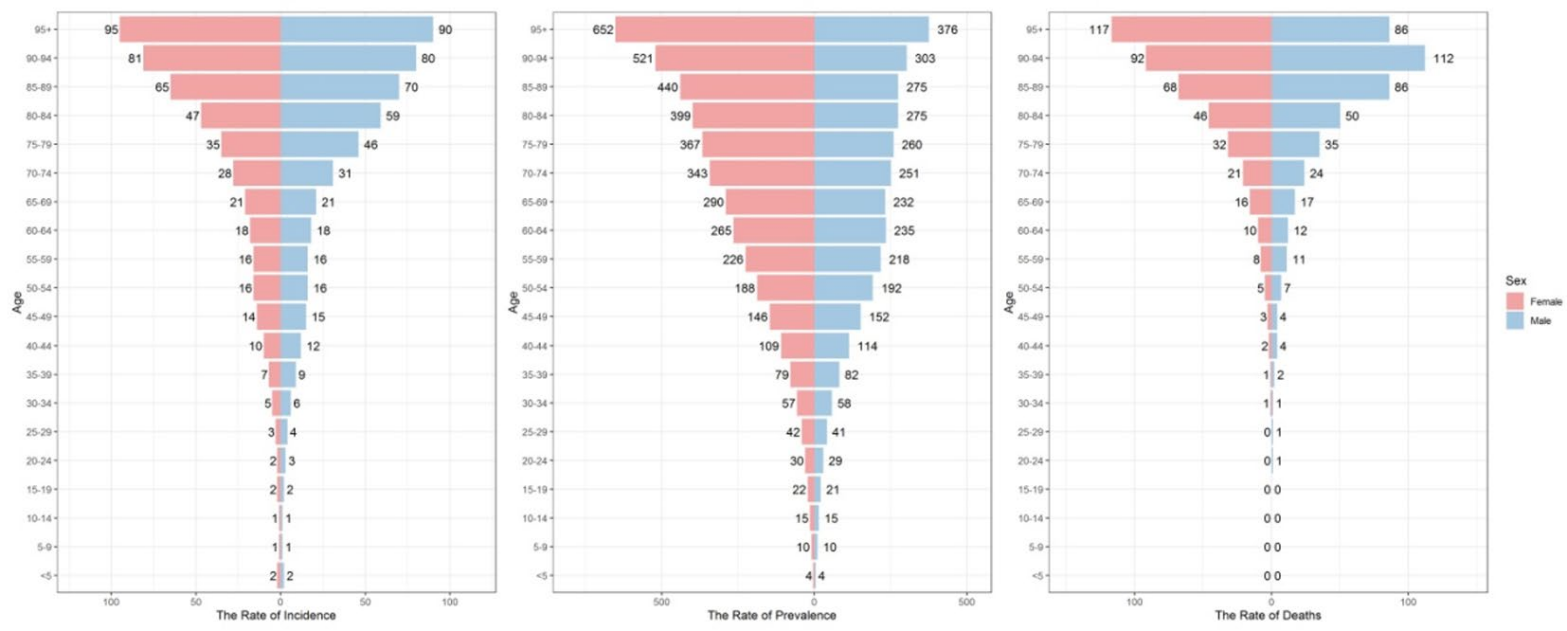
The rate of incidence, prevalence, and deaths due to SAH in Asia by sex in 2021.

Figure 2 shows that in 2021, the rates of SAH incidence in Asian women and men are generally similar, with Asian women having overall higher rates of SAH than men, and Asian women having slightly lower SAH mortality rates than men.

Overall, the incidence, prevalence, and mortality rates of SAH in Asia increased with age. For both men and women, the highest incidence and prevalence rates due to SAH are 95 years and over. The mortality rate occurs at ages 90–94 years for men and 95 years and over for women.

### Regional distribution of SAH in Asia

Table 2 shows that among the four regions in Asia, Southeast Asia had the highest ASPR of 110.62 per 100,000 population (95% UI 101.73, 120.43), while East Asia had the lowest ASPR of 70.06 per 100,000 population (95% UI 62.78, 77.98). In the High-income Asia Pacific region, the ASPR is high, at 193.60 per 100,000 population (95% UI 175.43, 212.12).

**Fig. 3:**
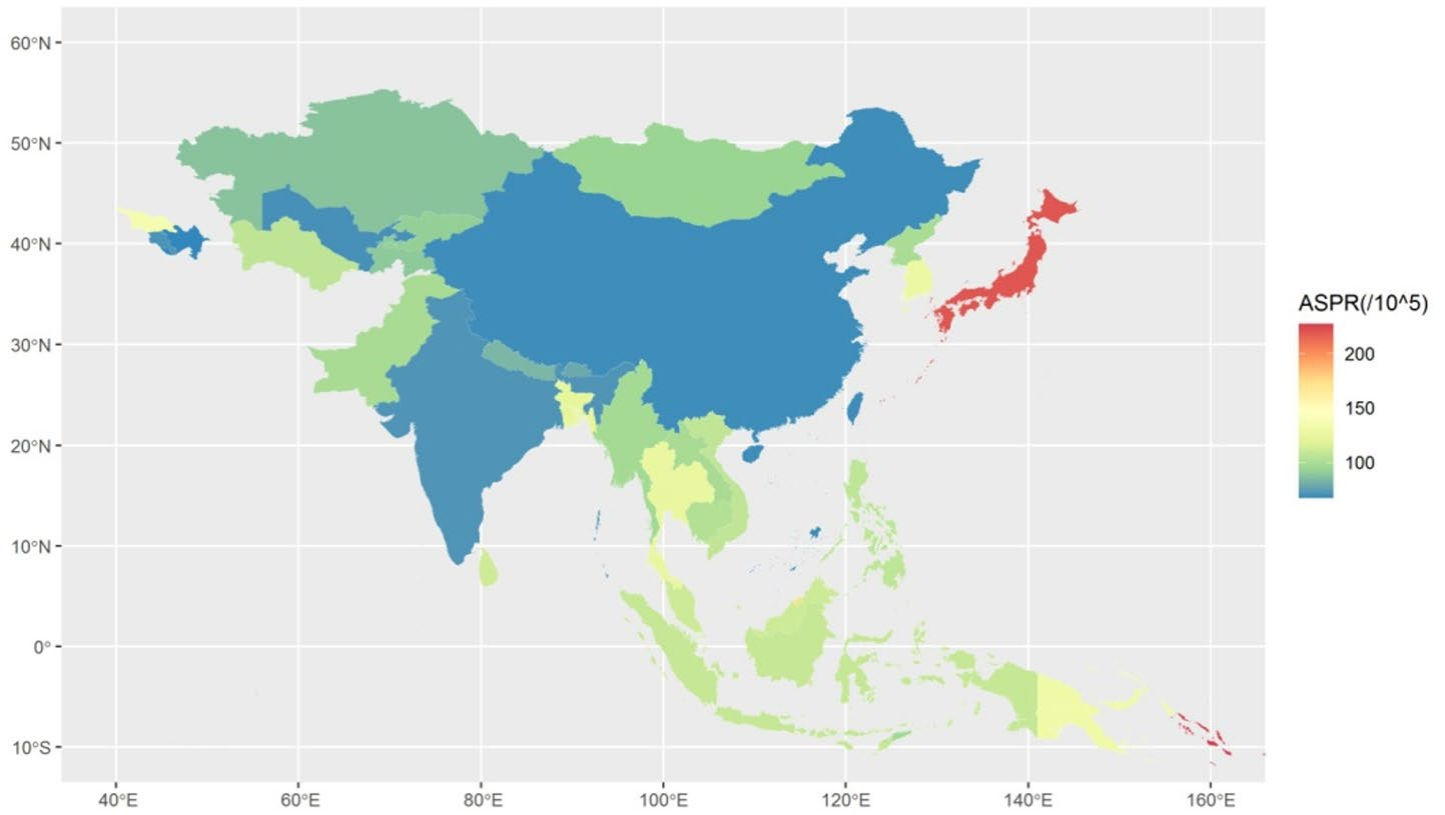
The map of age-standardized prevalence rate of SAH in Asian countries and regions.

Figure 3 shows the distribution of SAH in Asia in 2021. Table 2; Fig. 3 show that the Republic of Azerbaijan had the lowest age-standardized prevalence rate of 67.09 per 100,000 people (95% UI 61.95, 72.60), while the Solomon Islands had the highest age-standardized prevalence rate of 227.03 per 100,000 people (95% UI 213.74, 240.19).

**Table 2:** Number and ASR of the prevalence of SAH in different Asian countries in 2021.

Location		Number of cases (95% UI)	ASPR (95% UI) per 100,000
Asia		4597522.40(4163475.01, 5075403.49)	90.16(81.67, 99.22)
Central Asia	Republic of Armenia	2678.05 (2491.40, 2865.12)	71.14 (65.98, 76.17)
	Republic of Tajikistan	7154.53 (6618.82, 7685.61)	89.69 (82.68, 96.50)
	Georgia	6621.36 (6177.19, 7050.48)	136.10 (127.09, 144.83)
	Turkmenistan	2831.00 (2625.41, 3059.50)	106.44 (99.20, 113.84)
	Kyrgyz Republic	5225.13 (4886.49, 5587.83)	91.73 (84.64, 97.80)
	Republic of Azerbaijan	5536.35 (5113.21, 5916.82)	67.09 (61.95, 72.60)
	Mongolia	7885.31 (7284.45, 8524.59)	93.97(87.17, 101.53)
	Republic of Kazakhstan	17356.91 (16125.46, 18619.54)	87.86 (81.37, 94.49)
	Republic of Uzbekistan	22853.68 (21188.90, 24489.54)	70.07 (65.05, 75.12)
	Total	78142.32 (73006.78, 83491.46)	82.54 (77.09, 88.06)
South Asia	People’s Republic of Bangladesh	189240.81 (176671.42, 203827.17)	122.47 (114.19, 132.31)
	Republic of India	975214.96 (855887.76, 1105598.14)	71.80 (63.07, 81.19)
	Kingdom of Bhutan	549.87 (505.91, 595.24)	78.42 (72.16, 84.84)
	Federal Democratic Republic of Nepal	22404.24 (20693.71, 24275.50)	83.28 (76.73, 90.27)
	Islamic Republic of Pakistan	168288.13(148165.77, 189880.96)	98.96 (86.98, 112.63)
	Total	1355698.01(1203895.09, 1512996.08)	79.17 (70.58, 88.77)
Southeast Asia	Republic of the Philippines	105544.97 (94047.35, 118045.10)	106.64 (94.67, 119.65)
	Democratic Socialist Republic of Sri Lanka	29339.67 (27502.25, 31106.78)	112.28 (105.24, 119.06)
	Republic of the Union of Myanmar	53564.10(49834.02, 57329.71)	97.64(90.82, 104.40)
	Republic of Maldives	492.69(456.74, 529.19)	99.37 (92.52, 106.47)
	Kingdom of Cambodia	15314.42(14188.08, 16432.46)	102.64 (95.11, 109.79)
	Lao People’s Democratic Republic	5993.88 (5590.25, 6442.49)	99.12 (92.03, 106.28)
	Malaysia	35378.75(33143.74, 37644.08)	111.26 (104.02, 119.00)
	Republic of Indonesia	313471.21 (277711.59, 352552.11)	109.09(96.70, 122.68)
	Kingdom of Thailand	124120.52(116043.51, 131461.62)	126.06 (117.79, 133.79)
	Socialist Republic of Viet Nam	116554.55(109076.14, 124410.58)	107.41 (100.42, 114.73)
	Democratic Republic of Timor-Leste	983.55 (903.06, 1061.53)	96.75 (89.60, 103.82)
	Republic of Mauritius	2241.30(2091.18, 2389.78)	131.33 (122.54, 140.12)
	Republic of Seychelles	113.45 (105.40, 121.76)	93.05 (86.22, 100.16)
	Total	804234.79(736667.92, 876796.41)	110.62 (101.73, 120.43)
East Asia	People’s Republic of China	1323286.87(1176081.07, 1484052.12)	68.88 (61.53, 76.90)
	Republic of Korea	111725.26(105687.92, 117850.07)	127.76(120.90, 135.37)
	Taiwan (Province of China)	36836.34(34163.58, 39654.39)	108.90(101.54, 116.17)
	Total	1392547.97(1241282.77, 1558662.32)	70.06(62.78, 77.98)
High-income Asia Pacific	Japan	624486.73(552969.10, 709169.37)	219.60 (196.31, 244.74)
	Republic of Singapore	10868.99(10231.92, 11512.86)	130.49(122.69, 138.24)
	Brunei Darussalam	697.55(660.22, 738.56)	172.32 (162.82, 182.90)
	Republic of Korea	111725.26(105687.92, 117850.07)	127.76(120.90, 135.37)
	Total	747778.52(674045.57, 832435.30)	193.60(175.43, 212.12)

**Fig. 4:**
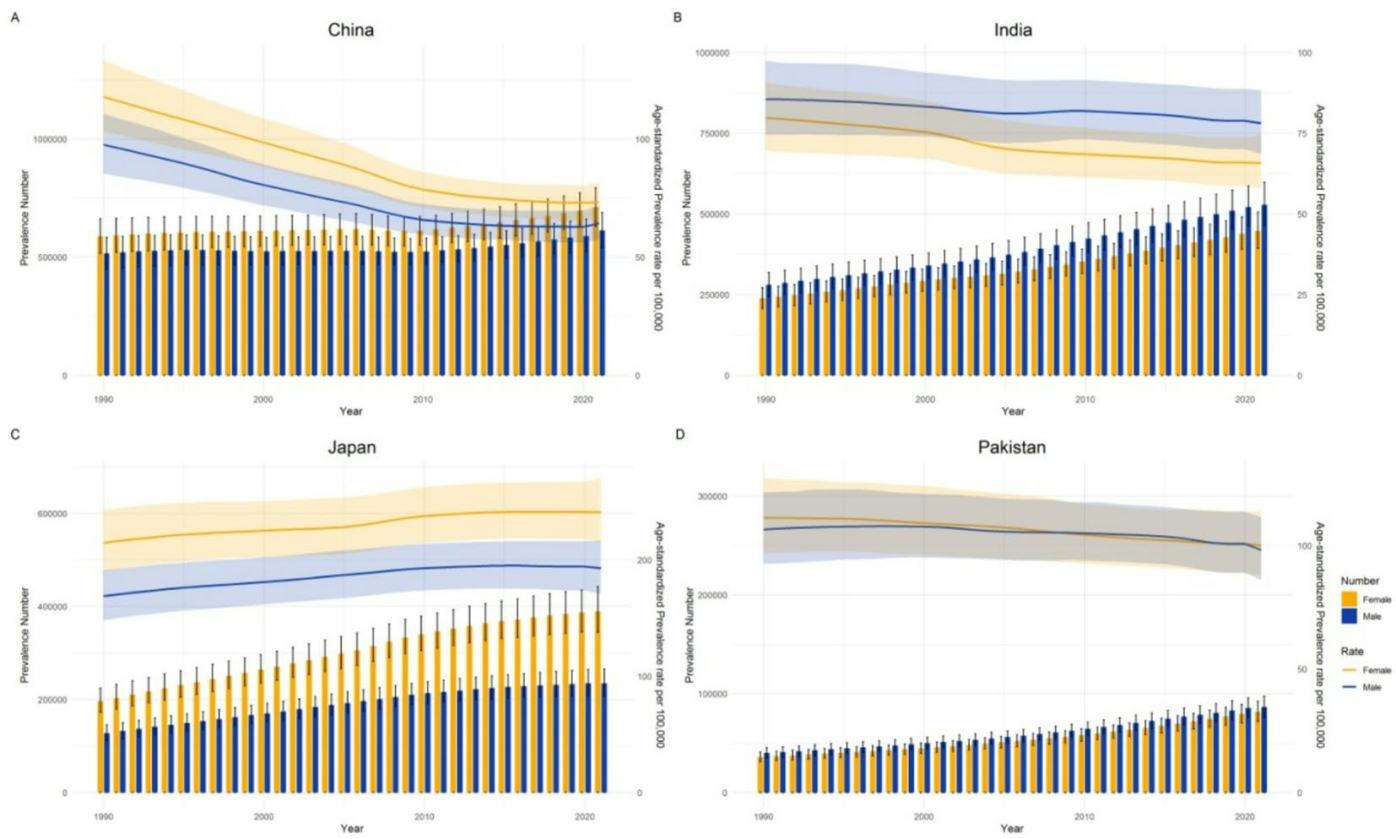
ASPR and prevalence number of SAH in China **(A)**, India **(B)**, Japan **(C)**, and Pakistan **(D)**, 1990–2021.

To better describe the temporal trends in SAH prevalence in Asia, four more typical countries were selected for further analysis. Both China and India are populous developing countries. In contrast, Pakistan, though also a developing country, has a smaller population. Japan, however, is a developed Asian nation with a relatively small population. We consider these four countries to be representative examples. Figure 4 shows that between 1990 and 2021, the prevalence of SAH showed an increasing trend in all four countries. In China, the ASPR showed a decreasing trend, whereas in Japan, it showed a slow increasing trend. On the other hand, in India and Pakistan, the trend in ASPR has fluctuated slightly but has generally shown a slow decreasing trend. In China and Japan, the ASPR and number of SAH cases are higher in women than in men, whereas in India, the opposite trend is observed. In Pakistan, the ASPR of SAH in males is not significantly different from that in females, and the number of cases in males is slightly higher than that in females.

### The Arima model predicts four age-standardized rates of SAH in Asia

**Fig. 5:**
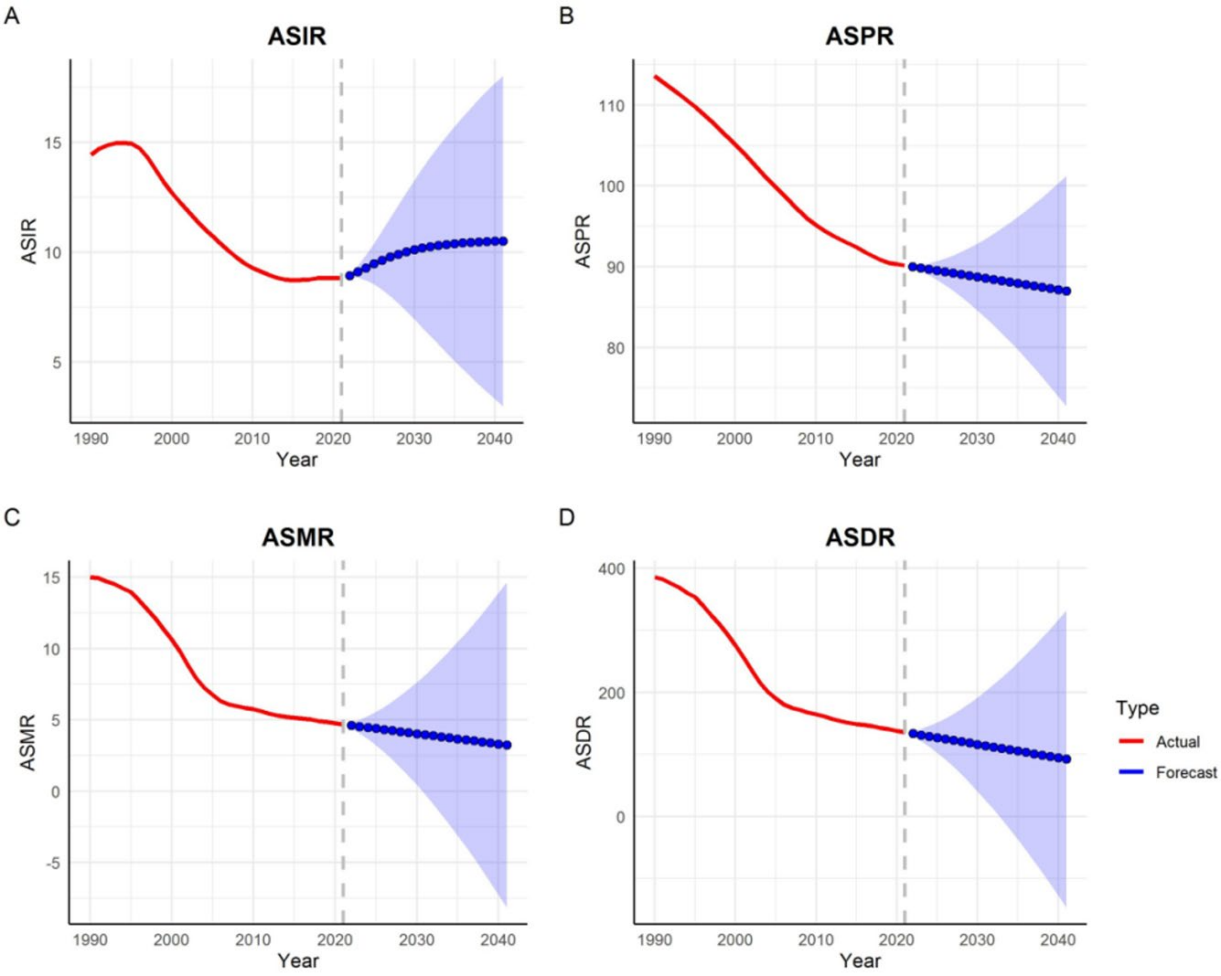
Forecast of SAH ASIR, ASPR, ASMR, and ASDR per 100,000 population from 2022 to 2041. **(A)** ASIR; **(B)** ASPR; **(C)** ASMR; **(D)** ASDR.

This study used the ARIMA model to predict the trends in the ASIR, ASPR, ASMR, and ASDR of SAH in Asia over the next 20 years. The optimal model parameters, along with their corresponding AIC, BIC, and Ljung–Box test p-values, are presented in Table 3. The Ljung-Box test showed that the p-values were all greater than 0.05, indicating that the original hypothesis could not be rejected and that all models exhibited white noise residuals, indicating stability and a good fit to the data. Figure 5 presents the anticipated ASIR, ASPR, ASMR, and ASDR per 100,000 individuals for the period spanning from 2022 to 2041.

As illustrated in Fig. 5, from 2022 to 2041, the ASPR, ASMR, and ASDR of SAH in Asia showed a decreasing trend, while the ASIR showed an increasing trend. By 2041, the projected age-standardized rates are as follows: incidence, 10.51 per 100,000; prevalence, 86.97 per 100,000; deaths, 3.24 per 100,000; DALYs, 92.38 per 100,000.

**Table 3:** Autoregressive integrated moving average (ARIMA) model parameters and their corresponding AIC and BIC for prediction of ASIR, ASPR, ASMR, and ASDR (per 100,000) for SAH for the next 20 years in asia. Based on the legend in Fig. 5, the red line represents the current data, while the blue line indicates the predicted data.

Measures	Parameters	AIC	BIC	Ljung–Box test *p*-value
Incidence	ARIMA(2,1,1)	−99.17	−93.43	0.699
Prevalence	ARIMA(1,2,0)	−73.37	−70.56	0.882
Deaths	ARIMA(0,2,0)	−46.07	−44.67	0.142
DALYs	ARIMA(0,2,0)	136.63	138.03	0.223

### Attributable risk factors for SAH dalys in 2021

**Fig. 6:**
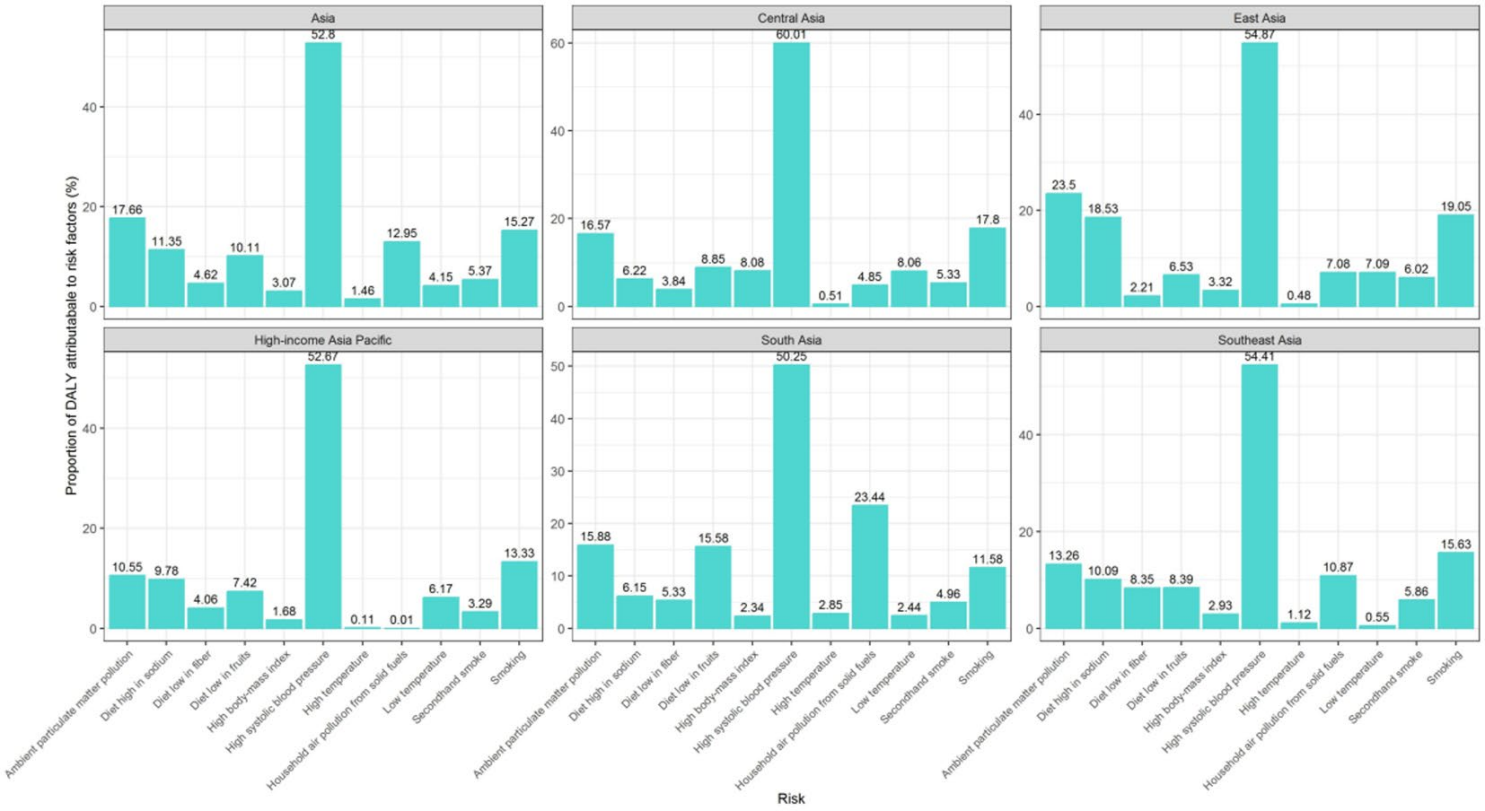
The proportion of DALYs of SAH attributable to 11 risk factors in 2021 by different regions of Asia.

In this study, an attributable risk analysis was conducted to evaluate the impact of 11 risk factors, including high systolic blood pressure, on SAH. As shown in the Fig. 6, high systolic blood pressure was the primary attributable risk factor in Asia and its various regions, accounting for over 50% of the total DALYs. The contributions of different risk factors varied across regions in Asia. In Asia, including Central Asia, East Asia, high-income Asia Pacific, and Southeast Asia, ambient particulate matter pollution and smoking are significant risk factors. And in South Asia, DALYs of SAH were more attributable to household air pollution from solid fuels (23.44%), ambient particulate matter pollution (15.88%), and diet low in fruits (15.58%), compared to other risk factors.

## Discussion

This study provides the first comprehensive analysis of the burden of SAH in Asia and applies the ARIMA model to forecast changes in the age-standardized rates of SAH.

The results indicate that between 1990 and 2021, the ASPR, ASMR, and ASDR of SAH in Asia decreased, and the ASIR of SAH in Asia first increased and then decreased, suggesting a reduction in the burden of SAH in Asia. This declining incidence trend is consistent with the findings of a previous systematic review and meta-analysis^4^. It is possible that this change was due to the improved management of hypertension and cessation of smoking. The global incidence of SAH has been shown to decrease by 7.1% for every 1 mmHg reduction in systolic blood pressure, 11.5% for every 1 mmHg reduction in diastolic blood pressure, and 2.4% for every 1% reduction in smoking prevalence^4^. Feigin et al. also showed that smoking and systolic blood pressure are the most important risk factors for SAH in the Asia-Pacific region^12^. Attributable risk analyses also showed that high systolic blood pressure was the primary attributable risk factor in Asia and its various regions. The decreased mortality is most likely due to advances in follow-up and treatment in recent years^13^. Although the ASIR, ASPR, ASMR, and ASDR for SAH in Asia have generally declined, the burden of public health expenditures associated with SAH remains significant. During the acute phase of SAH, patients require both diagnostic assessments and urgent treatment, which can be expensive. A significant proportion of SAH patients necessitate long-term rehabilitation following the acute phase, further increasing the burden on public health resources. The death and disability resulting from SAH deprive many families of their primary source of income, while imposing a heavy economic burden on society. This situation adversely affects productivity and overall societal development.

Among the four Asian regions, Southeast Asia had the highest ASIR, ASPR, ASMR, and ASDR values for SAH. In the high-income Asia Pacific, ASIR and ASPR are higher, and ASMR is lower compared to other regions in Asia. This may be attributed to the more adequate healthcare resources available in high-income Asia Pacific regions, including advanced neuroimaging equipment, specialized neurosurgeons, and neurointensive care units. While these advancements have contributed to reduced mortality rates, the burden of SAH remains significant as the population continues to age.

In 2021, the ASIR for SAH in Asian men and women was essentially similar, with the ASPR for men being smaller than that for women and the ASMR slightly larger than that for women. Women are thought to be at greater risk of developing spontaneous SAH than men^1^. A systematic review and meta-analysis suggested that this sex difference was associated with estrogen deficiency and postmenopausal collagen depletion in women, especially after the age of 50^14^. However, some studies have reported that the incidence of SAH is higher in men than in women^15^. This finding suggests that the disease burden of SAH in Asia is influenced by factors other than sex.

In Asia, the ASIR, ASPR, and ASMR of SAH increased with age, and the burden of SAH was concentrated in older age groups. Population aging is accelerating and has become a major global challenge, and its burden is expected to increase further^16^. This suggests that in the course of social development, more attention should be paid to the health status of the older patients and that health-care services should prioritize their needs.

Countries such as China and India have fairly large populations, leading to a high prevalence of SAH. China has the highest prevalence rate in Asia. China has long been a populous country. In 2019, China had 18% of the world’s population, with 164.5 million Chinese citizens aged 65 years and over, and 26 million aged 80 years or over^17^. There are significant regional differences in the burden of cardiovascular and cerebrovascular diseases with respect to national demographics and the varying levels of population aging in Asia^18^. China, India, Japan, and Pakistan showed an increasing trend in the prevalence of SAH from 1990 to 2021. In China, the ASPR showed a decreasing trend; in Japan, it showed an increasing trend, whereas in India and Pakistan, the ASPR showed little change. In China and Japan, the burden of SAH is greater in women than in men; in India, it is greater in men than in women; and in Pakistan, there is little difference in the burden of the disease between men and women. Differences in the burden of SAH among these countries are complex and influenced by many factors, including the environment, lifestyle, racial and ethnic differences, level of medical care, and socioeconomic development.

The ARIMA model projections for the next 20 years in Asia showed that while ASPR, ASMR, and ASDR will continue to decline, ASIR is expected to increase. This suggests that we need to be concerned about the incidence of SAH in Asia and proactively adopt preventive strategies to reduce the future burden of this disease.

This study was limited by inherent limitations associated with the global burden of disease research, including the quality and accessibility of raw data. Problems with the GBD persist, such as poor data quality and incomplete coverage of certain regions or countries. The lack of stroke and SAH registries in certain underdeveloped countries may result in an underestimation of data, leading to inaccuracies. The predictions of the ARIMA model may not consider the complexity of real-world scenarios, thus limiting predictive accuracy, especially in the case of unforeseen future crises or sudden shifts. Future research could attempt to develop new forecasting models to improve accuracy.

## Conclusion

This study analyzed the burden of SAH in Asia from 1990 to 2021 and forecasted potential trends for the period of 2022–2041. Currently, the disease burden of SAH in Asia remains high and poses a major threat to public health and economic development. There is a need to develop targeted policies and allocate health resources for the active prevention and treatment of SAH to reduce the burden of disease and mitigate the impact of SAH on countries, societies, and individuals.

## Data Availability

The datasets supporting the conclusions of this article are available in the Global Health Data Exchange (GHDx) query tool (https://ghdx.healthdata.org/gbd-2021).

## Abbreviations

SAH: Subarachnoid hemorrhage
GBD: Global Burden of Disease
DALYs: Disability-adjusted life years
YLD: Years lived with disability
YLL: Years of life lost
ASPR: Age-standardized prevalence rates
ARIMA: Autoregressive integrated moving average
ASIR: Age-standardized incidence rates
ASMR: Age-standardized mortality rates
ASDR: Age-standardized disability-adjusted life years rate
